# A Qualitative Assessment of the Content Validity of the ICECAP-A and EQ-5D-5L and Their Appropriateness for Use in Health Research

**DOI:** 10.1371/journal.pone.0085287

**Published:** 2013-12-19

**Authors:** Thomas Keeley, Hareth Al-Janabi, Paula Lorgelly, Joanna Coast

**Affiliations:** 1 MRC Midlands Hub for Trials Methodology Research, Health and Population Sciences, University of Birmingham, Birmingham, United Kingdom; 2 Health Economics Unit, Health and Population Science, University of Birmingham, Birmingham, United Kingdom; 3 Centre for Health Economics, Monash University, Victoria, Australia; Marienhospital Herne - University of Bochum, Germany

## Abstract

**Purpose:**

The ICECAP-A and EQ-5D-5L are two index measures appropriate for use in health research. Assessment of content validity allows understanding of whether a measure captures the most relevant and important aspects of a concept. This paper reports a qualitative assessment of the content validity and appropriateness for use of the eq-5D-5L and ICECAP-A measures, using novel methodology.

**Methods:**

In-depth semi-structured interviews were conducted with research professionals in the UK and Australia. Informants were purposively sampled based on their professional role. Data were analysed in an iterative, thematic and constant comparative manner. A two stage investigation - *the* *comparative* *direct* *approach* - was developed to address the methodological challenges of the content validity research and allow rigorous assessment.

**Results:**

Informants viewed the ICECAP-A as an assessment of the broader determinants of quality of life, but lacking in assessment of health-related determinants. The eq-5D-5L was viewed as offering good coverage of health determinants, but as lacking in assessment of these broader determinants. Informants held some concerns about the content or wording of the Self-care, Pain/Discomfort and Anxiety/Depression items (EQ-5D-5L) and the Enjoyment, Achievement and attachment items (ICECAP-A).

**Conclusion:**

Using rigorous qualitative methodology the results suggest that the ICECAP-A and EQ-5D-5L hold acceptable levels of content validity and are appropriate for use in health research. This work adds expert opinion to the emerging body of research using patients and public to validate these measures.

## Introduction

The ICECAP-A is a relatively new index measure. Its theoretical grounding is in Amartya Sen’s work on functioning and capability [1–4] which encourages a broad evaluative space through a focus on what a person is able to do and who they are able to be, rather than what they actually do and who they become [5]. The descriptive system of the ICECAP-A capability measure, formed through in depth interviews with the general public, defines quality of life as consisting of: Stability, Attachment, Autonomy, Achievement and Enjoyment [6]. The measure assesses capability by asking whether a person “can” or is “able” to achieve particular states. The ICECAP-A measure was developed in the UK and is already being used within health research in a number of countries including Australia and Canada.

The EQ-5D-5L is a generic preference based outcome measure, which measures health-related quality of life. The descriptive system remains the same as the original EQ-5D-3L measure: mobility, self-care, usual activities, pain and discomfort and anxiety and depression [7], with the number of response options in each dimension increased from three to five. This aims to increase the responsiveness of the measure and reduce ceiling effects [8–12]. The EQ-5D-5L has been translated into 97 languages and work to elicit value sets has begun in a number of countries [13]. 

Content validity is the extent to which a descriptive system of a measure “represents the most relevant and important aspects of a concept in the context of a given measurement application” [14]_p.743_. Assessment of the content coverage of a measure allows understanding about inferences that can be drawn from the results of a measure. Here, content validity concerns whether the ICECAP-A and the EQ-5D-5L assess the most important and relevant attributes of quality of life and health-related quality of life, respectively. 

When measuring a non-tangible concept, the questions of what is relevant and what should be measured is a longstanding methodological challenge in content validation [15,16]. This challenge is particularly important when attempting to validate the content of quality of life and health-related quality of life measures, where no universally accepted definition exists. An analysis that seeks to answer the question “does the measure sample the important and relevant dimensions of a construct?” requires clarity regarding these important and relevant dimensions. Therefore, qualitative analyses of the content validity of a measure should seek to assess not only the opinions of the measure under consideration, but also the informant’s conceptualisation of the concept the measure seeks to assess. Having an understanding of the informant’s conceptualisation of the concept creates the potential for a fuller understanding of the opinions of the measure and a firmer conclusion as to the content validity of the measure.

Rigorous and transparent qualitative methodology, absent from much of the research associated with quality of life measure validation, provides a suitable way for assessing content validity [17–19]. Qualitative content validation can be completed with the public, patients and experts in relevant fields acting as informants. Patients have first hand, personal experience of both the concept and how it might be affected by different situations. They are in a position to provide an insider’s – emic – perspective. Experts, such as clinicians or researchers can provide an outsider – etic – perspective. They have the advantage of observing both a number of individuals in different situations and how the construct of interest manifests itself in different individuals [14]. While there is an emerging quantitative evidence base of validity amongst patients and public for both the EQ-5D-5L [8,20,21] and ICECAP-A [22], qualitative work using experts as informants is needed to allow triangulation of data from different perspectives and different methodologies [14,19].

This paper reports a qualitative assessment of the content validity of the EQ-5D-5L and ICECAP-A measures and their appropriateness for use in health research. 

## Methods

### Informant selection

Informants were recruited from the UK and Australia to provide an international assessment of these measures. Using maximum variation sampling [23] informants were purposively selected from: 1) clinical and public health trial experts (“trialists”); 2) medical doctors involved in research; 3) researchers with regular participant contact (“frontline researchers”); and 4) health economists working within a trial setting. These groups were selected to sample across a broad spectrum of professional experiences and research perspectives. 

Invitation emails stating the aim of the research, the potential burden upon the informant and the ethical approval gained for the study were sent to potential informants. Snowball recruitment, whereby previously interviewed informants were asked to give recommendations of other potential informants, was used to recruit three frontline researchers. Recruitment was stopped when data saturation was identified. 

### Ethics statement

The study protocol, which included participants providing written consent prior to the interview, was approved by the University of Birmingham Science, Technology, Engineering and Mathematics Ethical Review Committee (ERN_11-0575). 

### The interview and analysis process

Interviews were broadly partitioned into two parts using a semi-structured topic guide designed to facilitate breadth and depth of discussion. A two stage investigation of content validity, termed the comparative direct approach, was developed based on this partitioning. The development of this approach sought to address the methodological challenge discussed above by providing a useful structure within which a thematic analysis, grounded in the data could be completed. 

The first part of the interview assessed informants’ understanding of quality of life as a concept, what influenced this understanding and how the diseases they worked with professionally affected their perspective. Content mapping and mining questions were used to encourage breadth and depth [24]. Differences between quality of life and health-related quality of life were explored. When analysing the data from this first part of the interview the informants’ descriptions of quality of life were *compared* by the researcher with the descriptive systems of the ICECAP-A and EQ-5D-5L. This comparison enabled identification of the parts of a measure’s descriptive system that the informants felt were important.

In the second part of the interview, informants were presented with copies of ICECAP-A and EQ-5D-5L, one after the other, in random order. Informants were encouraged to *directly* discuss the measure’s content coverage and its appropriateness for use in their research area. Informants were asked to think back to how *they* defined quality of life and health-related quality of life in the first part of the interview, and assess whether they felt the measure covered *their* conceptualisation. Data from the second part of the interview was analysed to assess informants’ opinions about how well the measures covered their own conceptualisation of quality of life. Using the informant’s conceptualisation of quality of life as a reference point facilitated the analysis: knowing what the informants understood quality of life to be and what dimensions they held to be important and relevant, allowed greater understanding of their opinions of the measures. 

All interviews were conducted by TK, who was not involved in the development of either of the measures under consideration. Interviews were recorded, transcribed verbatim and transcripts were coded using a hierarchical and flexible coding structure. The first version of this coding structure was formed during the completion of the first analysis batch and was therefore grounded in the data. This coding structure allowed data to be coded under broader themes as well as under more focused categories referring to a specific topic. An iterative, constant comparative, thematic analysis of the transcripts was completed [17,25,26]. Transcripts were analysed in four successive batches. This analysis allowed descriptive and explanatory accounts to be formed and comparisons to be drawn between informants. The iterative nature of the analysis allowed themes which were identified in earlier batches to be analysed and developed in later batches of the analysis. Themes in the data were identified by the authors and developed through the use of the flexible coding structure. TK led the coding and analysis of the data and work was checked regularly by HAJ, PL and JC for consistency and accuracy. The qualitative data analysis computer package ATLAS.ti was used to assist this analysis. Verbatim quotes from informants have been selected to illustrate how informants’ accounts were linked to emerging themes. Ellipses (…) were used to denote missing speech. ‘Umm’, ‘err’ and repetitions of words, which do not add meaning, were removed without the use of ellipsis.

## Findings

In this section the informants’ conceptualisations of quality of life and its determinants are presented. The overall perceptions of the measures, followed by an item by item breakdown of the results of the researcher led *comparative* analysis and the informant led *direct* analysis, for the ICECAP-A and EQ-5D-5L are presented in turn. 

### Interviews

Interviews, lasting between 45 and 90 minutes, were conducted with 17 informants in the UK and Australia between February and September 2012 (see Table 1 for informant characteristics). Interviews were conducted at the informants’ place of work. None of the informants were involved in the development of either the ICECAP-A or EQ-5D-5L measures, nor did they hold any professional relationship with TK. Data saturation was identified at interview 14. Three additional interviews were conducted to check saturation and ensure adequate numbers were sampled from each professional role. 

**Table 1:** Informant characteristics.

	Number interviewed (n=17)
**Sex**	
Male	7
Female	10
**Location**	
Australia	8
UK	9
**QoL measure experience of use**	
EQ-5D	15
ICECAP	5
**Professional role**	
Frontline researcher	4
Trialist	6
Health economist	3
Research doctor	4

### Informant conceptualisation of quality of life and health-related quality of life

Physical health was identified by an overwhelming majority of informants as an important determinant of both an individual’s health-related quality of life and quality of life. Pain was identified as a particularly pervasive determinant and for informants who worked in cancer research the side-effects of treatment were a particular concern. Psychological health was also seen as having a notable impact on quality of life. Many informants discussed how psychological problems, such as depression, can stem from a physical condition. 

*I still see health as important…I think when someone has got ill health...it is quite a big determinant*. [Frontline researcher, Australia]

Informant discussions indicated they differentiated between quality of life and health-related quality of life, with the later closely related to physical and psychological health. Quality of life was viewed as a broader construct and the terms “big picture”, “multi-dimensional” and “broad” were frequently used. Although physical and psychological health were considered major determinants, informants recognised that people can have adequate or even high quality lives, despite being in poor health states. 

*…all you see is ill health and states that you don’t want to get into, but there are people that get into those states and have a fantastic time.* [Trialist, UK]

Relationships with friends and family were viewed as important due to the enjoyment and support they can provide. Informants often described the importance of friends and family from the perspective of losing loved ones. The ability of an individual to lead their normal life was discussed by a sizable minority of informants. Informants attached importance to individuals being able to fulfil the roles within society which they value. 

*…that they are able to perform social roles that they would normally perform [is important].* [Trialist, Australia]

### ICECAP-A

Informants viewed the ICECAP-A as a broad assessment of quality of life, appropriate for use in the research fields in which they worked. It was viewed as a short, uncomplicated measure, suitable for a busy research environment:

*It is a lovely length...because…you don’t have the time to spend with a long questionnaire*. [Frontline researcher, UK]

Most informants felt that the ICECAP-A captured the important determinants of quality of life as described in part one of the interview. The notable exception was that informants felt that it did not directly assess health, which informants had identified as an important determinant.

*It is just how far away from health it gets I suppose...I think it is just the distance from health*. [Health economist, UK]

*Yeh I guess this one is more general...and focuses mostly on the emotional*. [Trialist, Australia]

Most informants felt the measure was patient-focused, while a very small minority, who were more likely to be trialists than frontline researchers or doctors, felt the subject matter was too sensitive for patients. There was a consensus that the measure would be favoured in addition to, rather than as a replacement for, existing health-related quality of life measures. Informants felt that a measure that maintained a focus on health-related quality of life was also required. 

*I wouldn*’*t see it as a replacement for an EQ-5D, but it would certainly complement an EQ-5D type instrument*. [Health economist, UK]

A small number of informants showed a level of cognitive struggle in understanding the capability wording of the ICECAP-A measure. For these informants there was some concern about whether the wording would be understood by participants in the studies.

*I don’t like the “I am able” or “I can”, I don’t know, it feels as if in some way you are the person with the control , so I CAN have a lot if I want to I can have a lot of love and friendship*. [Trialist, UK]

#### Stability

Prior to viewing the measure in the first part of the interview, informants identified stability in life as an important determinant of quality of life. Living with fear and uncertainty due to a physical condition or illness and the concern that unemployment due to illness can cause, was identified by a number of informants. 

*You get frightened of taking your medicine. You get frightened of going to sleep, in case you don’t wake up*. [Research doctor, Australia]

Upon seeing the measure, there was broad acceptance that the Stability item was relevant to the assessment of quality of life and would be influenced by both health and non-health factors. Some informants recognised that the item was assessing a construct that they had previously identified as important.

*...it makes sense because...the patients I see are very palliative and they don’t have a lot of time. But you can still be settled and secure with months to live*. [Frontline researcher, UK]

#### Attachment

Prior to viewing the measure, informants identified the ability to function in a social context as an important consideration both for the enjoyment and support it provides. The significance to people suffering from illnesses of achieving social contact and the limiting effect that illnesses can have upon ability to achieve social contact was discussed at length.

*And in the last year of his life, he died by the cancer, he said...this has been the best year of my life, because until this moment I never realised how loved I’ve been*. [Trialist, UK]

Upon considering the measure concern was raised by a small number of informants about the perceived sensitivity of the subject, while the majority recognised Attachment as being both relevant and seldom assessed. 

*Well things like love, friendship and support. It is all that thing around social connectiveness and support and intimacy. We as a research group are very interested in that in people with HIV* [Research doctor, Australia] 

#### Autonomy

A small number of informants discussed independence as a dimension of quality of life prior to seeing the measure. Informants focused on the ability to do day-to-day activities that were often closely linked with mobility.

*...they can’t get down to the shops to do their shopping…It is hard, they have got to think about is it feasible to do something that they want to, based on how mobile they are*. [Trialist, Australia]

In comparison to the limited discussion prior to viewing the measure, most informants identified the Autonomy item as being of central importance to the assessment of quality of life. There was a consistent view that it was particularly important to elderly people. 

*...especially with older people that independence is hugely important to them, and that’s one of the depressing things for them when they lose that independence I think*. [Frontline researcher, UK]

#### Achievement

The influence upon quality of life of being able to achieve and attain personal goals was not discussed by many informants prior to viewing the ICECAP-A. However, gaining a sense of achievement through work and being able to look back at life with a sense of achievement were discussed briefly by a small number of informants. 

*...i think he [young cancer sufferer*]* has kind of condensed it all to “Yeah, I am 25 and I have achieved everything I want”...and he is perfectly sane in what he is saying*. [Frontline researcher, UK]

On seeing the measure, Achievement was thought to be more relevant to younger rather than older people. The use of the word “progress” in the item was questioned. For some it focused on the area of paid employment, while those who worked in cancer noted that oncology patients could misunderstand the question as assessing the progress of their cancer. The item was considered by a number of informants as being too broad and some questioned whether the top item was really achievable.

*I don’t think that I can achieve and progress in all aspects of my life, I would love to be able to. BUT*. [Trialist, UK]

#### Enjoyment

Enjoyment was discussed as an important influence on quality of life from the perspective of people with illnesses or disabilities enjoying life in spite of their condition. It was normally identified through providing examples, rather than stating explicitly that enjoyment was a construct of quality of life.

*You have people that have an enormously great quality of life who can’t walk anywhere...because they have this great social structure and play cards all day*. [Trialist, Australia]

On considering the item, informants were split between those who felt Enjoyment was important and relevant, and those who did not. For those who felt Enjoyment was not relevant, a motivating factor appeared to be that the item was too broad to be relevant. 

*What do you mean by enjoyment and pleasure?...I suppose not vague, but possibly ambiguous*. [Health economist, UK]

### EQ-5D-5L

Informants viewed the EQ-5D-5L measure as a simple and straight forward measure of health. The length and simplicity of the measure was viewed positively and a number of informants noted that the language used was appropriate.

*I think the great beauty of this is that you can do this in two minutes flat*. [Trialist, UK]

Informants viewed it as a measure of health state, which captured the determinants of health-related quality of life they had described previously. However informants noted that it did not capture the broad spectrum of quality of life they had described. 

*...that one is more broadly health*. [Research doctor, Australia]

*...it is not capturing how they feel about their life. It’s, they are not saying “I have a good life” or not...This one is what you can do and what problems do you have*. [Trialist, UK]

There was broad recognition of the usefulness of the measure in health research. This appeared to be motivated partly by awareness of a strong precedent of use of the EuroQol measure and its recognition by funding and rationing bodies.

*It is hard to beat the EuroQol in terms of NICE guidance and everything that is out there already*. [Health economist, Australia]

Informants who had previously used EQ-5D-3L thought that the increase in levels would improve the ability of the measure to record change in health state, reducing the “ceiling effect” which existed in the old measure and making it more attractive. Enthusiasm was shown for the new version.

*I think I would prefer it to the EuroQol that we are using now*. [Frontline researcher, Australia]

#### Mobility

In the prior discussion informants identified the ability to be mobile, as well as the ability to move upper and lower limbs as important determinants of quality of life. Mobility was not valued for itself, rather for allowing individuals the independence to access their normal everyday life.

*...it is independence, it is to do with mobility, it’s the getting to the shops, being able to do what you want to do, when you can do it...* [Trialist, Australia]

Disagreement existed about whether the item fully assessed mobility. Informants noted that the item assessed a persons’ ability to walk, not their ability to be independently mobile. This was considered to limit the scope of the item.

*...that’s just about walking, whereas people can be independently mobile in a wheel chair, they can actually have quite a high quality of life*. [Trialist, Australia]

#### Self-care

Prior to viewing the measure, no informant directly identified self-care as an important determinant of quality of life.

Upon seeing the measure informants felt the Self-care item was narrow, and arbitrary. Many felt it should be considered as part of usual activities, rather than as a dimension in and of itself.

*...as a sort of category of assessment...it is only one action, like making a cup of tea. It is a bit arbitrary really*. [Trialist, UK]

#### Usual activities

Informants discussed the importance of people being able to complete normal activities, such as going to work and having social contact. In the broad conceptualisation of quality of life offered by informants in their prior discussions, a large number of the non-health determinants appeared to relate to usual activities. 

*...in terms of their participation, that they are not able to do things that they normally do...whether it is looking after the grandchildren or cooking meals or something like that*. [Trialist, Australia]

Informants considered the Usual Activities item to be broad and noted the need for the clarifying statement. While there was hesitance in the language used referring to the breadth of the item, only a few informants directly stated that breadth was a problem.

*Whereas usual activity, work, home work, leisure is massively broad*. [Trialist, UK]

#### Pain/Discomfort

Pain was identified as a particularly important, almost pervasive, health-related influence on quality of life. Informants with clinical training discussed how pain could be managed to reduce its influence on quality of life.

*Nothing worse for quality of life in many ways than chronic discomfort and pain*. [Research doctor, Australia] 

The Pain/Discomfort item was noted as being an important aspect to measure. Some concern existed about the phrasing of the question, with a small number of informants noting that the item assesses two distinct dimensions: pain or discomfort.

*You got to wonder why you’d bother asking pain or discomfort. Wouldn’t you ask one, because they are so different*... [Trialist, Australia]

#### Anxiety/Depression

The effect of the psychological state upon quality of life was identified by a large number of informants and was thought to be influenced heavily by physical health. Worry, concern, fear, anxiety and depression were identified as important psychological determinants.

*Depression is a frequent co-morbidity of severe physical illness*. [Researcher doctor, Australia]

Informants thought the Anxiety/Depression item was very relevant. There was concern about the stigma attached to the word “depression” and how this might influence participants’ answers. The use of the words “depression” and “anxiety” as a summary for psychological health was felt to lack scope.

*...anxious and depressed...people don’t like that word depression. You know,* “don’t tell me that”... [Frontline research, Australia]

## Discussion

Informants considered the ICECAP-A to offer comprehensive measurement of the broad construct of quality of life they described, while lacking a direct assessment of health. The EQ-5D-5L was viewed as offering good coverage of health-related quality of life, while lacking assessment of the broader determinants of quality of life. This assessment is largely in line with the aims of each measure: the EQ-5D-5L is designed to measure the health determinants of quality of life, while the ICECAP-A capability measure’s theoretical grounding focuses on wellbeing more broadly defined. These results therefore suggest that the ICECAP-A and EQ-5D-5L measures hold acceptable levels of content validity. 

The item by item analysis showed that informants had concerns about some items in each measure. The content of the EQ-5D-5L Self-Care item was not viewed as relevant, while the restricted content of the Mobility item was questioned. In the ICECAP-A the content relevance of the Autonomy and Achievement items was thought to be age dependent. Other concerns pertained to the phrasing of some items (Achievement, Pain/Discomfort, Anxiety/Depression) or the potential for items to upset participants due to its subject matter (Attachment). 

Both EQ-5D-5L and ICECAP-A were viewed as short, simple and easy to use, which is in line with findings from qualitative research with the general population for the EQ-5D-5L [7] and ICECAP-A [27]. The increase in levels of the EQ-5D-5L was expected to improve responsiveness and reduce “ceiling effects”, this been shown in early quantitative assessments [11,12]. Concerns over interpretation of the capability wording in the ICECAP-A measure should be considered in light of research that found the general public were able to understand and answer the questions [27]. Use of the ICECAP-A in addition to the EQ-5D-5L rather than as a replacement was viewed as a positive step in assessing quality of life.

The rigorous qualitative methodology used is a notable strength of this study, which importantly adds an expert perspective to the validity portfolios of both measures. Although the number of informants interviewed was relatively small in absolute terms, importantly, it was sufficient to achieve saturation; there was however a slight oversampling of informants who work in cancer research. It was not possible to assess the effect that the informants familiarity with existing quality of life and health-related quality of life measures had on their opinions of these measures, this is particularly true for the findings of the EQ-5D-5L where a number of informants had used the original 3 level version. It was not possible to assess whether the order in which the measures were viewed influenced responses, however the random order of presentation should have controlled for this to some degree. 

Further qualitative and quantitative research, providing assessments of the content validity, construct validity and responsiveness of both measures in different clinical areas will be important. In line with the objectives of this research, to assess the validity and acceptability of these measures in a health setting, the informants used were health research professionals. This may have led to an increased focus on the physical health determinants of quality of life. Both the EQ-5D-5L and ICECAP-A may be of use in social care, public health and mental health research. Further qualitative research examining the content validity of these measures with patients and researchers in these areas is highlighted as an important area of research. 

In conclusion, although there are concerns about specific content of some individual items, this study offers evidence of the content validity and appropriateness of both measures in a trial context in the UK. Informants viewed the ICECAP-A as an assessment of the broader determinants of quality of life; while the EQ-5D-5L was viewed as offering good coverage of health determinants of quality of life. This is largely in line with the objectives of both measures. This research adds an expert perspective to the emerging validity portfolios of these measures and in doing so allows greater confidence in the validity of these measures.
